# Combined prognostic nutritional index and albumin-bilirubin grade to predict the postoperative prognosis of HBV-associated hepatocellular carcinoma patients

**DOI:** 10.1038/s41598-021-94035-5

**Published:** 2021-07-16

**Authors:** Xie Liang, Xu Liangliang, Wang Peng, Yan Tao, Zhang Jinfu, Zhang Ming, Xu Mingqing

**Affiliations:** 1 Department of Liver Surgery, West China Hospital, Sichuan University, No. 37 Guo Xue Xiang, Chengdu, 610041 China; 2 Department of Hepatobiliary Surgery (2), The Affiliated Hospital of North Sichuan Medical College, Nanchong, China

**Keywords:** Cancer, Oral diseases, Liver diseases

## Abstract

This study aims to evaluate the predictive value of the prognostic nutritional index (PNI) and albumin-bilirubin grade (ALBI) for the postoperative prognosis of hepatitis B virus-associated hepatocellular carcinoma (HBV-HCC) patients undergoing radical hepatectomy (RH). Besides, we seek to identify novel prognosis indicators for HBV-HCC patients. Between April 2009 and March 2015, this work enrolled 868 patients diagnosed with HBV-HCC and undergoing RH in the Liver Surgery Department, West China Hospital, Sichuan University (WCHSU). The basic information, laboratory examination indicators, pathological reports, and follow-up data of patients were included. SPSS 22.0 statistical software was used for statistical data analyses. Platelet (PLT), alpha-fetoprotein (AFP), maximum diameter (max-D), number of tumors (Number), degree of differentiation (DD), Microvascular invasion situation (MVI), satellite focus situation (SF), PNI, and ALBI were the independent risk factors for both overall survival (OS) and disease-free survival (DFS) of HBV-HCC patients undergoing RH. Taking PNI = 46 and ALBI = − 2.80 as cut-off values, the OS and DFS of the PNI-high group were significantly higher than those of the PNI-low group. Meanwhile, the OS and DFS of the ALBI-low group were significantly higher than those of the ALBI-high group; the OS and DFS of the PNI-high + ALBI-low group were significantly higher than those of the PNI-low + ALBI-high group. Xie prognostic index (XPI) was the independent risk factor for both OS and DFS of HBV-HCC patients undergoing RH. The OS and DFS of the XPI-high group were significantly higher than those of the XPI-low group. This paper reveals that preoperative PNI and ALBI can predict the OS and DFS of HBV-HCC patients undergoing RH. Their impact on the prognosis of HBV-HCC patients is insignificant, however, it cannot be ignored. XPI can precisely predict the prognosis of HBV-HCC patients undergoing RH, nonetheless, its effect requires additional research for validation.

## Introduction

Hepatocellular carcinoma (HCC) is the 5th most prevalent malignancy and the 4th most common cause of cancer-related mortalities across the globe^1,2^. An estimated 840,000 newly diagnosed HCC cases are annually reported across the globe; approximately half of these are in China^3,4^. About 100 million people are infected with the hepatitis B virus (HBV) in China; this is associated with 70% to 90% of HCC patients^5^.

Radical hepatectomy (RH) is a primary treatment for hepatitis B virus (HBV)-associated hepatocellular carcinoma (HBV-HCC) patients. It is the most common treatment in China because of the limited availability of donor organs and expensive medical cost which complicates liver transplantation^6^. With the rapid development of surgery, RH has a 5-year overall survival (OS) rate of only 60–70%^7^. Therefore, there is an urgent need to improve the OS of HBV-HCC patients. HBV-HCC prognosis is linked to several factors, including the tumor size, alpha-fetoprotein (AFP), disease stage, and vascular invasion^8^. Besides, the preoperative nutritional status, immune and liver functions are important factors affecting the OS of HBV-HCC patients^9,10^.

Hepatitis B is a chronic consumptive infectious disease, while HCC is a malignant tumor with a profound effect on whole-body function. Therefore, their occurrence and progress influence the nutritional status and immune function of HBV-HCC patients. In 1984, Onodera suggested a prognostic nutritional index (PNI) as a nutritional prognostic indicator for patients diagnosed with gastrointestinal diseases^11^. PNI is easily calculated with a formula that includes the serum albumin level and lymphocyte count. At present, it is believed that PNI effectively reflects the nutritional and immune status of patients^12,13^. Current studies reveal that PNI is significantly related to HCC prognosis, however, contrasting voices have also been reported^10^.

Albumin-bilirubin grade (ALBI) is an indicator of liver function; it should only be calculated with serum albumin and total bilirubin^14^. As one of the most severe liver diseases, HBV-HCC inevitably influences the liver function of patients, then reflects ALBI changes.

A correlation between PNI, ALBI, and HCC prognosis^10–12,15^ has been substantially studied, nevertheless, the small sample size has yielded significantly different results. Meanwhile, limited studies have been conducted on HBV-HCC patients undergoing RH, and no study has been reported on the combination of PNI and ALBI as a prognostic prediction model of HBV-HCC patients undergoing RH. As such, studies using a larger sample size are essential to guide clinical treatment.

## Subjects and methods

### Subjects

Between April 2009 and March 2015, we enrolled 868 patients diagnosed with HBV-HCC undergoing RH in the Liver Surgery Department, West China Hospital, Sichuan University (WCHSU). The age of the patients ranged from 12 to 82 years, with an average age of 50.53 ± 12.04 years; 727 (76.7%) males, average age 50.74 ± 11.89 years; 141 (14.9%) females, average age 49.44 ± 12.82 years).

Inclusion criteria: (1) RH was only performed in our hospital, and HCC was confirmed by pathology; RH was defined as no residual tumor and a negative resection (R0) margin based on the pathology examination; (2) no evidence of extrahepatic metastasis; (3) HBsAg was positive. Exclusion criteria: (1) Patients with malnutrition caused by other severe diseases; (2) Patients received other preoperative anticancer treatments; (3) Patients with infectious diseases (urinary tract infection, respiratory tract infection, etc.) or other chronic wasting diseases; (4) Patients with other malignancy; (5) Without complete clinical or follow-up data.

### Methods

#### Research indicators

Gender, age, admission time, contact information, and Barcelona Clinic Liver Cancer Stage (BCLC) of all patients were obtained from the medical records. Blood routine test and liver function examination were performed to record platelets (PLT), total lymphocytes (TLC), white blood cells (WBC) and albumin (ALB), total bilirubin (TBil), Aspartate Aminotransferase (AST), and Alanine Aminotransferase (ALT). Also, HBV-DNA, HBV Surface Antigen (HBsAg), alpha-fetoprotein (AFP) were recorded. In addition to AFP^16^, the cut-off value of the above indicators was set based on the normal reference range provided by the WCHSU laboratory. All blood indicators were collected within one week before the operation.

The maximum diameter (max-D) and the number of tumors, Ishak stage of paracancerous tissue (Ishak)^17^, liver capsule invasion situation (LCI), degree of differentiation (DD), Microvascular invasion situation (MVI), and satellite focus situation (SF) were recorded based on the pathological report of patients.

The Edmondson-Steiner classification criteria were used to evaluate the differentiation grade^18^. The diagnostic standard of MVI and SF was based on “the evidence-based practice guidelines for standardized pathological diagnosis of primary liver cancer in China: 2015 update”^19^.

#### Follow-up

After discharge, regular telephone or outpatient follow-up commenced one month after the operation, and every three months within three years, and every half year thereafter. The endpoints of this study were 1 -, 3 -, and 5-year disease-free survival (DFS) and OS. Disease-free survival (DFS) is the period between the date of RH and the date of recurrence confirmation. Overall survival (OS) refers to the period between the date of RH and the date of death or the last follow-up. The last follow-up time was August 31, 2018.

#### Nutritional status indicator

Prognostic nutritional index (PNI) was used to evaluate the nutritional status was evaluated. Its formula was PNI = 10 × ALB (g/dL) + 0.005 × TLC (per mm^3^)^9^ = ALB (g/L) + 5 × TLC (10^9^/L). Based on previous studies^10,15^, lower PNI value worsens the nutritional status of patients.

#### Liver function indicator

Albumin-bilirubin grade (ALBI) was used to evaluate the liver function. Its formula was ALBI = log_10_ TBil (μmol/L) × 0.66 − ALB (g/L) × 0.085^14^. According to previous studies^14,20^, higher ALBI value worsens the liver function of patients.

#### Statistical analysis

All statistical data were entered and analyzed using SPSS 22.0 software (IBM SPSS Inc., Chicago, IL, USA) and MedCalc 20.0 software (MedCalc Software, Mariakerke, Belgium). The mean ± standard deviation was used to describe the continuous data which obeyed normal distribution. Student T-test, chi-square, or Pearson correlation analysis was appropriately used to compare related variables. The receiver operating characteristic (ROC) curve was drawn to determine the best cut-off value^21^. The survival curves were plotted using the Kaplan–Meier method and compared using the log-rank test. Further, the Cox multivariate analysis identified independent risk factors. *P* < 0.05 was considered statistically significant.

### Ethics approval and consent

This study was approved by the Clinical Research Ethics Committee of West China Hospital, Chengdu, China (IRB number: FWA00009482IRBIORG0004190), and followed the guidelines outlined in the declaration of Helsinki. The study has received the informed consents from all patients or from parent and/or legal guardian if participants are under 18.

## Results

### The cut-off value of PNI and ALBI

**Figure 1:**
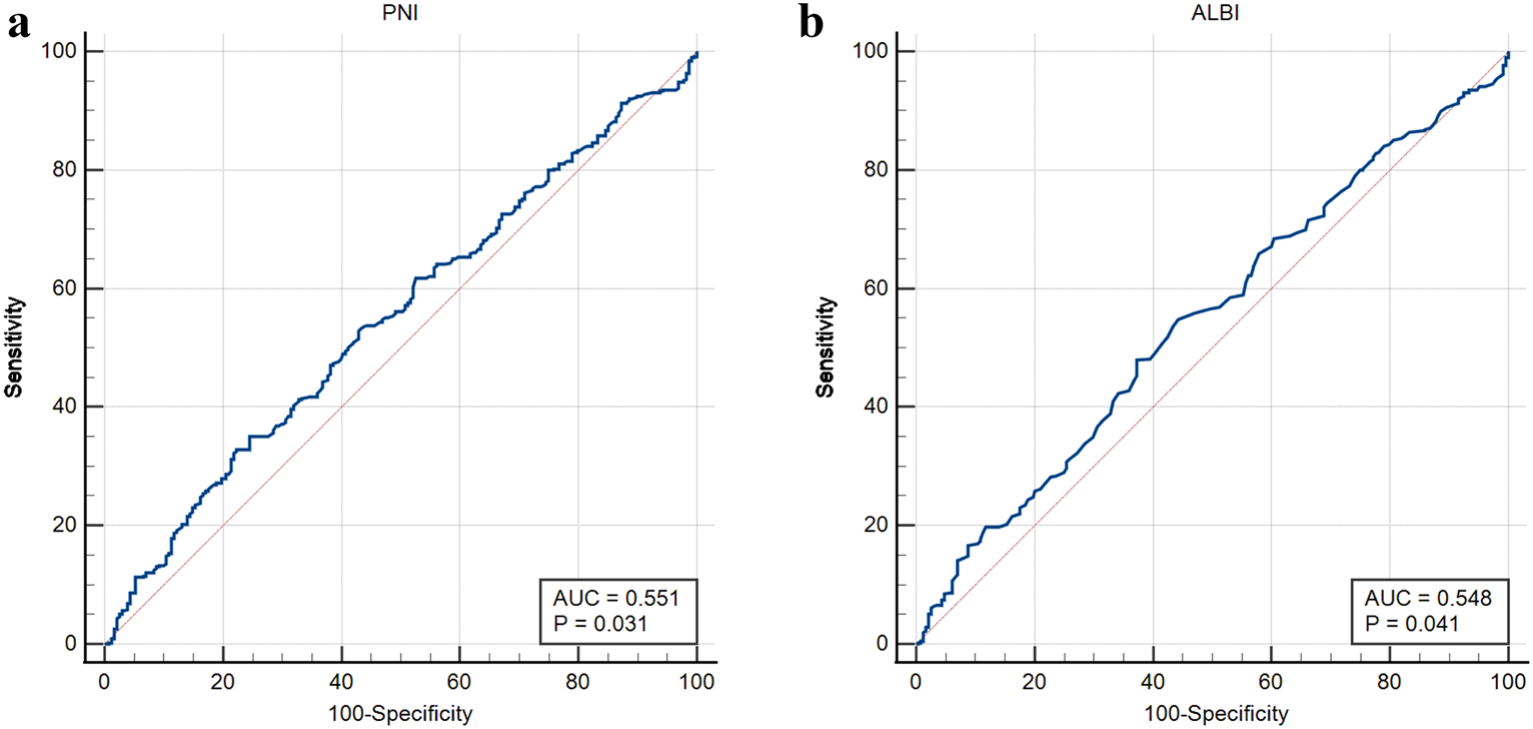
ROC curve for determination of the cut-off value of PNI (a) and ALBI (b) for predicting 5-year OS.

According to Fig. 1a, the optimal cut-off value of PNI was 46, sensitivity = 0.776, specificity = 0.328, corresponding to maximum Youden index (= 0.105) for predicting 5-year OS in ROC analysis. Therefore, the patients were divided into the PNI-high (PNI > 46) group or the PNI-low (PNI ≤ 46) group.

Based on Fig. 1b, the optimal cut-off value of ALBI was -2.80, sensitivity = 0.549, specificity = 0.557, corresponding to maximum Youden index (= 0.106) for predicting 5-year OS in ROC analysis. The patients were thus divided into the ALBI-high (ALBI > -2.80) group or the ALBI-low (PNI ≤ -2.80) group.

### Correlation between PNI or ALBI and clinical or pathological indicators of HBV-HCC patients undergoing RH

The correlation analysis between PNI, ALBI, and the general condition, hematological qualitative indicators of the patients is shown in Table 1. The difference in age and PLT between the PNI-high group and the PNI-low group was statistically significant (all P < 0.05). Similarly, TLC, WBC, ALB, TBil, AST, HBV-DNA, and AFP levels were significantly different between the PNI-high group and PNI-low group (all P < 0.01). The WBC was markedly different between the ALBI-high group and the ALBI-low group (P < 0.05). Further, TLC, ALB, TBil, AST, ALT, HBV-DNA, and AFP levels were substantially different between ALBI-high group and ALBI-low group (all P < 0.01).

**Table 1:** The correlation analysis between PNI, ALBI and the general condition, hematological qualitative indicators.

Indicators	n	PNI		P	Cases	ALBI		P
		≤ 46	> 46			> − 2.80	≤ − 2.80	
**Gender**								
Male	727	194 (26.7%)	533 (73.3%)	0.776	727	352 (48.4%)	375 (51.6%)	0.910
Female	141	36 (25.5%)	105 (74.5%)		141	69 (48.9%)	72 (51.1%)	
**Age (years)**								
< 60	645	163 (25.3%)	482 (74.7%)	0.230	645	303 (47.0%)	342 (53.0%)	0.174
≥ 60	214	63(29.4%)	151 (70.6%)		214	112 (52.3%)	102 (47.7%)	
**BCLC**								
0-A	745	191 (25.6%)	554 (74.4%)	0.158	745	356 (47.8%)	389 (52.2%)	0.298
B-D	123	39 (31.7%)	84 (68.3%)		123	65 (52.8%)	58 (47.2%)	
**PLT (10**^**9**^**/L)**								
< 100	268	85 (31.7%)	183 (68.3%)	0.020*	268	131 (48.9%)	137 (51.1%)	0.882
≥ 100	600	145 (24.2%)	455 (75.8%)		600	290 (48.3%)	310 (51.7%)	
**TLC (10**^**9**^**/L)**								
< 1.1	191	102 (53.4%)	89 (46.6%)	< 0.001**	191	110 (57.6%)	81 (42.4%)	0.004**
≥ 1.1	677	128 (18.9%)	549 (81.1%)		677	311 (45.9%)	366 (54.1%)	
**WBC (10**^**9**^**/L)**								
< 4.0	114	47 (41.2%)	67 (58.8%)	< 0.001**	114	61 (53.5%)	53 (46.5%)	0.038*
≥ 4.0	429	95 (22.1%)	334 (77.9%)		429	183 (42.7%)	246 (53.3%)	
**ALB (g/L)**								
< 40	297	201 (67.7%)	96 (32.3%)	< 0.001**	297	289 (97.3%)	8 (2.7%)	< 0.001**
≥ 40	571	29 (5.1%)	542 (94.9%)		571	132 (23.1%)	439 (76.9%)	
**TBil (μmol/L)**								
< 28	831	211 (25.4%)	620 (74.6%)	≤ 0.001**	831	390 (46.9%)	441 (53.1%)	≤ 0.001**
≥ 28	37	19 (51.4%)	18 (48.6%)		37	31 (83.8%)	6 (16.2%)	
**AST (IU/L)**								
< 40	456	90 (19.7%)	366 (80.3%)	≤ 0.001**	456	189 (41.4%)	267 (58.6%)	< 0.001**
≥ 40	412	140 (34.0%)	272 (66.0%)		412	232 (56.3%)	180 (43.7%)	
**ALT (IU/L)**								
< 50	565	141 (25.0%)	424 (75.0%)	0.160	565	250 (44.2%)	315 (55.8%)	0.001**
≥ 50	303	89 (29.4%)	214 (70.6%)		303	171 (56.4%)	132 (43.6%)	
**HBV-DNA (cps/ml)**								
< 1000	315	67 (21.3%)	248 (78.7%)	0.001**	315	127 (40.3%)	188 (59.7%)	≤ 0.001**
≥ 1000	421	138 (32.8%)	283 (67.2%)		421	230 (54.6%)	191 (45.4%)	
**AFP (ng/ml)**								
< 400	526	123 (23.4%)	403 (76.6%)	0.009**	526	236 (44.9%)	290 (55.1%)	0.006**
≥ 400	340	107 (31.5%)	233 (68.5%)		340	185 (54.4%)	155 (45.6%)	

*The correlation was significant in 0.05 layer (two tailed), similarly hereinafter.

**The correlation was significant in 0.01 layer (two tailed), similarly hereinafter.

The correlation analysis between PNI, ALBI, and pathological qualitative indicators of the patients is shown in Table 2. Levels of max-D and DD were markedly different between PNI-high group and PNI-low group, as well as between the ALBI-high group and ALBI-low group (all P < 0.05).

**Table 2:** The correlation analysis between PNI, ALBI and pathological qualitative indicators.

Indicators	n	PNI		*P*	Cases	ALBI		*P*
		≤ 46	> 46			> − 2.80	≤ − 2.80	
**max-D (cm)**								
< 5	387	84 (21.7%)	303 (78.3%)	0.005**	387	170 (43.9%)	217 (56.1%)	0.014*
≥ 5	476	144 (30.3%)	332 (69.7%)		476	249 (52.3%)	227 (47.4%)	
**Number**								
1	719	188 (26.1%)	531 (73.9%)	0.607	719	343 (47.4%)	376 (52.3%)	0.302
≥ 2	149	42 (28.2%)	107 (71.8%)		149	78 (52.3%)	71 (47.4%)	
**Ishak (score)**								
0–4	297	77 (25.9%)	220 (74.1%)	0.783	297	134 (45.1%)	163 (54.9%)	0.150
5–6	571	153 (26.8%)	418 (73.2%)		571	287 (50.3%)	284 (49.7%)	
**LCI**								
No	254	62 (24.4%)	192 (75.6%)	0.350	254	127 (50.0%)	127 (50.0%)	0.721
Yes	467	129 (27.6%)	338 (72.4%)		467	227 (48.6%)	240 (51.4%)	
**DD (grade)**								
1–2	501	120 (24.0%)	381 (76.0%)	0.047*	501	228 (45.5%)	273 (54.5%)	0.039*
3	367	110 (30.0%)	257 (70.0%)		367	193 (52.6%)	174 (47.4%)	
**MVI**								
No	642	167 (26.0%)	475 (74.0%)	0.585	642	305 (47.5%)	337 (52.5%)	0.323
Yes	226	63 (27.9%)	163 (72.1%)		226	116 (51.3%)	110 (48.7%)	
**SF**								
No	754	201 (26.7%)	553 (73.3%)	0.783	754	363 (48.1%)	391 (51.9%)	0.586
Yes	114	29 (25.4%)	85 (74.6%)		114	58 (50.9%)	56 (49.1%)	

### Univariate and multivariate analyses of the prognostic factors of the HBV-HCC patients undergoing RH

PNI, ALBI, and clinical and pathological factors were evaluated by univariate and multivariate analyses to determine the risk factors for DFS and OS. As shown in Table 3, and through univariate analysis, BCLC, PLT, AST, HBV-DNA, AFP, max-D, Number, LCI, DD, MVI, SF, PNI, and ALBI were significant prognostic factors for both OS and DFS (all *P* < 0.05).

**Table 3:** Prognostic factors for 1-, 3-, 5-year(s) DFS and OS by univariate analysis.

Indicators	n	DFS			*P*	OS			*P*
		1-year	3-years	5-years		1-year	3-years	5-years	
**Gender**									
Male	727	66.4%	43.1%	31.3%	0.179	87.5%	64.1%	48.8%	0.405
Female	141	65.2%	49.4%	39.7%		82.3%	64.6%	56.5%	
**Age (years)**									
< 60	645	63.7%	43.2%	31.9%	0.086	85.3%	63.1%	49.7%	0.381
≥ 60	214	74.8%	47.8%	35.9%		91.1%	68.0%	52.2%	
**BCLC**									
0-A	745	69.1%	47.7%	35.8%	< 0.001**	87.8%	67.6%	53.5%	< 0.001**
B-D	123	48.8%	22.5%	13.4%		79.7%	43.4%	27.7%	
**PLT (109/L)**									
< 100	268	71.6%	50.5%	36.0%	0.023*	91.0%	73.2%	58.7%	< 0.001**
≥ 100	600	63.8%	41.3%	31.2%		84.7%	60.2%	46.1%	
**TLC (109/L)**									
< 1.1	191	64.8%	44.5%	32.9%	0.947	86.9%	60.5%	48.1%	0.809
≥ 1.1	677	66.6%	44.0%	32.6%		86.6%	65.3%	50.5%	
**WBC (109/L)**									
< 4.0	114	75.4%	54.3%	39.4%	0.064	91.2%	71.0%	52.6%	0.177
≥ 4.0	429	65.0%	42.5%	31.2%		86.9%	64.3%	48.7%	
**ALB (g/L)**									
< 40	297	66.7%	42.8%	30.5%	0.655	85.2%	61.7%	46.0%	0.059
≥ 40	571	66.0%	44.8%	34.0%		87.4%	65.5%	52.2%	
**TBil (μmol/L)**									
< 28	831	66.4%	43.6%	33.1%	0.967	86.8%	64.2%	49.5%	0.433
≥ 28	37	62.2%	56.8%	23.9%		83.8%	64.8%	61.2%	
**AST (IU/L)**									
< 40	456	71.7%	50.6%	38.3%	< 0.001**	90.6%	70.2%	56.3%	< 0.001**
≥ 40	412	60.1%	37.0%	26.6%		82.3%	57.6%	43.1%	
**ALT (IU/L)**									
< 50	565	66.3%	45.1%	33.9%	0.303	86.9%	66.0%	50.6%	0.253
≥ 50	303	66.0%	42.3%	30.5%		86.1%	60.8%	48.7%	
**HBV-DNA (cps/ml)**									
< 1000	315	69.8%	50.9%	40.9%	0.001**	89.8%	69.6%	57.8%	0.001**
≥ 1000	421	62.4%	37.8%	25.0%		84.6%	61.5%	44.7%	
**AFP (ng/ml)**									
< 400	526	75.7%	52.2%	37.9%	< 0.001**	92.6%	72.5%	58.0%	< 0.001**
≥ 400	340	51.7%	31.5%	24.3%		77.4%	51.1%	37.4%	
**max-D (cm)**									
< 5	387	78.8%	58.3%	42.3%	< 0.001**	94.3%	77.4%	64.6%	< 0.001**
≥ 5	476	56.5%	32.9%	24.8%		80.9%	53.7%	38.7%	
**Number**									
1	719	68.7%	48.1%	36.2%	< 0.001**	87.5%	67.2%	53.4%	< 0.001**
≥ 2	149	54.4%	25.1%	15.4%		82.6%	49.6%	32.5%	
**Ishak (score)**									
0–4	297	64.6%	43.0%	33.3%	0.614	85.9%	62.0%	49.7%	0.539
5–6	571	67.0%	44.7%	32.2%		87.0%	65.4%	50.2%	
**LCI**									
No	254	72.3%	47.5%	34.8%	0.012*	90.2%	70.4%	51.8%	0.003**
Yes	467	58.5%	39.3%	32.1%		82.7%	57.0%	44.0%	
**DD (grade)**									
1–2	501	73.1%	49.9%	36.0%	< 0.001**	91.6%	72.1%	56.3%	< 0.001**
3	367	56.9%	36.1%	28.1%		79.8%	53.3%	41.4%	
**MVI**									
No	642	71.9%	49.5%	37.3%	< 0.001**	89.4%	69.4%	55.6%	< 0.001**
Yes	226	50.0%	29.0%	19.4%		78.8%	49.4%	33.9%	
**SF**									
No	754	69.1%	47.7%	35.7%	< 0.001**	88.3%	68.5%	53.6%	< 0.001**
Yes	114	47.4%	20.9%	12.5%		75.4%	35.5%	25.4%	
**PNI**									
≤ 46	230	62.6%	38.4%	23.9%	0.007**	84.3%	57.1%	38.8%	< 0.001**
> 46	638	67.5%	46.2%	35.8%		87.5%	66.8%	54.2%	
**ALBI**									
>− 2.8	421	63.6%	40.9%	27.6%	0.013*	83.4%	58.4%	44.4%	< 0.001**
≤− 2.8	447	68.7%	47.1%	37.7%		89.7%	69.7%	55.3%	

Pearson correlation analysis showed a strong negative correlation between PNI and ALBI (r = − 0.810, *P* < 0.01); it could not be simultaneously placed into a Cox proportional hazards model, hence Cox proportional hazards models were established respectively. After stepwise regression, BCLC, AST, HBV-DNA, and LCI were not introduced into the final multivariate regression models. After adjustment of hazard ratio (HR) via multivariate analysis, PNI (in Model 1) and ALBI (in Model 2) independently predicted the OS and DFS (all *P* < 0.05) (Table 4).

**Table 4:** Prognostic models for DFS and OS by Cox multivariate regression analysis. (Model 1: variables that were independent risk factors of PNI were included; Model 2: variables that were independent risk factors of ALBI were included).

Indicators	Model 1				Model 2			
	OS		DFS		OS		DFS	
	HR (95% CI)	P	HR (95% CI)	P	HR (95% CI)	P	HR (95% CI)	P
PLT	1.411 (1.125–1.770)	0.003	1.207 (1.002–1.454)	0.047	1.376 (1.098–1.724)	0.006	1.270 (1.058–1.525)	0.010
AFP	1.591 (1.301–1.944)	< 0.001			1.595 (1.305–1.949)	< 0.001		
max-D	1.672 (1.345–2.078)	< 0.001	1.563 (1.310–1.864)	< 0.001	1.682 (1.354–2.090)	< 0.001		
Number	1.369 (1.070–1.751)	0.012	1.566 (1.264–1.940)	< 0.001	1.364 (1.067–1.743)	0.013	1.540 (1.245–1.904)	< 0.001
DD	1.466 (1.200–1.790)	< 0.001	1.280 (1.079–1.517)	< 0.001	1.465 (1.200–1.788)	< 0.001	1.268 (1.071–1.502)	0.006
MVI	1.525 (1.234–1.885)	< 0.001	1.491 (1.238–1.795)	< 0.001	1.515 (1.226–1.871)	< 0.001	1.612 (1.342–1.937)	< 0.001
SF	1.924 (1.487–2.489)	< 0.001	1.633 (1.291–2.064)	< 0.001	1.907 (1.475–2.466)	< 0.001	1.690 (1.339–2.133)	< 0.001
PNI	1.314 (1.063–1.623)	0.011	1.220 (1.015–1.466)	0.034				
ALBI					1.272 (1.045–1.549)	0.017	1.191 (1.010–1.404)	0.038

### OS and DFS-survival curve based on PNI or ALBI

**Figure 2:**
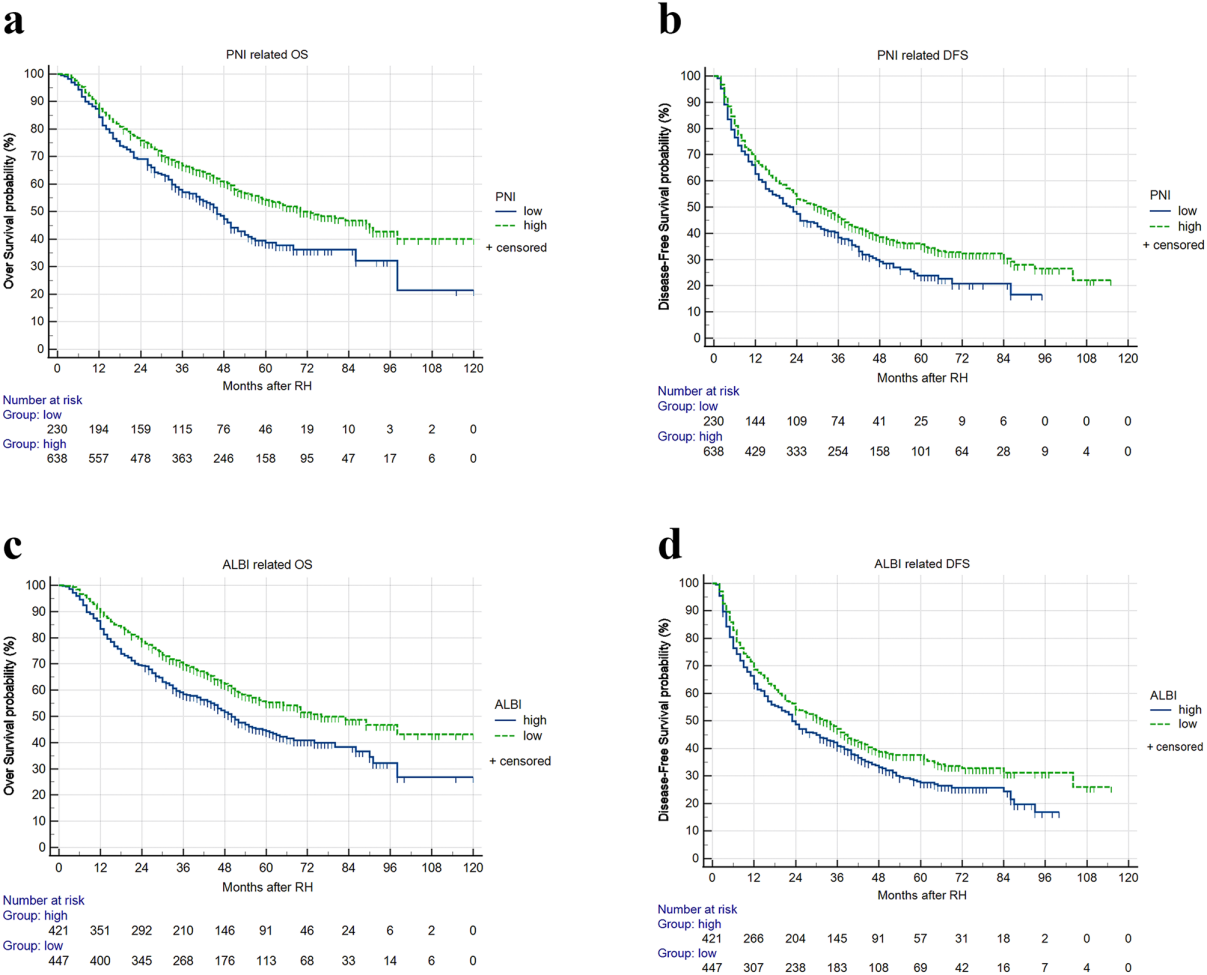
Relationship between PNI or ALBI and DFS or OS of HBV-HCC patients undergoing RH. (**a**) OS of patients with PNI > 46 was higher thant hose with PNI ≤ 46 (P < 0.01). (**b**) DFS of patients with PNI > 46 was higher than those with PNI ≤ 46 (P < 0.01). (**c**) OS of patients with ALBI ≤ -2.8 was higher than those with ALBI > -2.8 (P < 0.01). (**d**) DFS of patients with ALBI ≤ -2.8 was higher than those with ALBI > -2.8 (P < 0.05).

In contrast with the PNI-low group, the PNI-high group had significantly higher 1-, 3-, and 5-year OS (87.5% vs 84.3%, 66.8% vs 57.1% and 54.2% vs 38.8%, respectively, *P* < 0.01) (Fig. 2a) and 1-, 3-, and 5-year DFS (67.5% vs 62.6% and 46.2% vs 38.4%, 35.8% and 23.9%, respectively, *P* < 0.01) (Fig. 2b). This indicates that PNI is positively correlated with survival rate in HBV-HCC patients undergoing RH.

In contrast with the ALBI-low group, the ALBI-high group had significantly lower 1-, 3-, and 5-year OS (83.4% vs 89.7%, 58.4% vs 69.7% and 44.4% vs 55.3%, respectively, *P* < 0.01) (Fig. 2c) and 1-, 3-, and 5-year DFS (63.6% vs 68.7% and 40.9% vs 47.1%, 27.6% and 37.7%, respectively, *P* < 0.05) (Fig. 2d). This suggests that ALBI is negatively correlated with the survival rate in HBV-HCC patients undergoing RH.

### The prognostic value of the PNI-ALBI combination and Xie prognostic index in HBV-HCC patients undergoing RH

**Figure 3:**
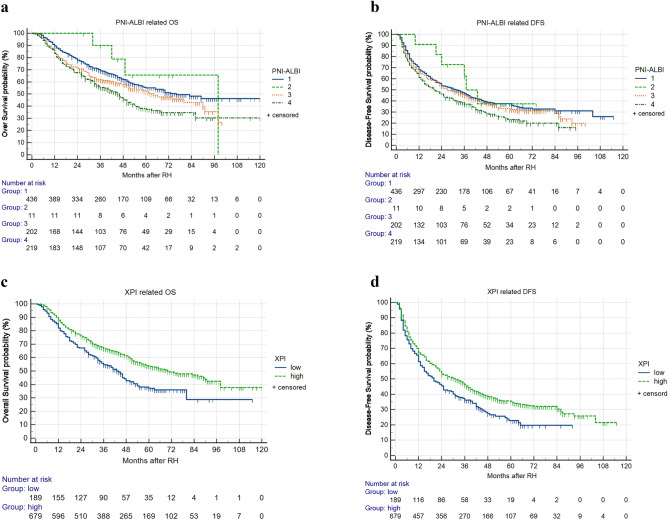
Relationship between PNI-ALBI or XPI and DFS or OS of HBV-HCC patients undergoing RH. (**a**) Relationship between PNI-ALBI and OS. (**b**) Relationship between PNI-ALBI and DFS. (**c**) OS of patients with XPI ≤ 75 was lower than those with XPI > 75 (P < 0.01). (**d**) DFS of patients with XPI ≤ 75 was lower than those with XPI > 75 (P < 0. 01).

Since PNI and ALBI could not simultaneously predict OS and DFS in the same model, PNI = 46 and ALBI = − 2.8 were taken as cut-off values; besides, PNI and ALBI were combined to divide patients into four groups i.e., group 1—PNI-high + ALBI-low; group 2—PNI-low + ALBI-low group; group 3—PNI-high + ALBI-high; group 4—PNI-low + ALBI-high. The OS (P < 0.01, Fig. 3a) and DFS (P < 0.05, Fig. 3b) of group 1 were significantly higher than those of group 4. However, there was no significant difference between one of them with group 2 or group 3.

The above results are not ideal. The two formulas were integrated to form a formula that includes ALB, TLC, and TBil; it simultaneously reflects the nutritional immunity status and liver function of HBV-HCC patients. First, a weight coefficient “K” (the ratio of ALB’s coefficient in PNI formula to ALBI’s coefficient in ALBI formula) was identified to balance the proportion of ALB in the two formulas:

$${\text{K}} = {{{\text{ALB}}} \mathord{\left/ {\vphantom {{{\text{ALB}}} {\left( { - 0.0{85} \times {\text{ALB}}} \right) = - {2}00/{17}}}} \right. \kern-\nulldelimiterspace} {\left( { - 0.0{85} \times {\text{ALB}}} \right) = - {2}00/{17}}}$$

Then, the new index “Xie prognostic index (XPI)” temporarily is named and defined as:

$$\begin{aligned} {\text{XPI}} & = {\text{PNI}} + {\text{K}} \times {\text{ALBI}} \\ & {\text{ = ALB}}\;\left( {{\text{g}}/{\text{L}}} \right) + 5 \times {\text{TLC}}\;\left( {10^{9} /{\text{L}}} \right) + \left( { - 200/17} \right) \times \left[ { \, \log_{10} {\text{TBil}}\;\left( {\mu {\text{mol}}/{\text{L}}} \right) \times 0.66 - {\text{ALB}}\;\left( {{\text{g}}/{\text{L}}} \right) \times 0.085} \right] \\ & = 2 \times {\text{ALB}}\;\left( {{\text{g}}/{\text{L}}} \right) + 5 \times {\text{TLC}}\;\left( {10^{9} /{\text{L}}} \right) - (132/17) \times \left[ { \, \log_{10} {\text{TBil}}\;\left( {\mu {\text{mol}}/{\text{L}}} \right)} \right] \\ \end{aligned}$$

**Figure 4:**
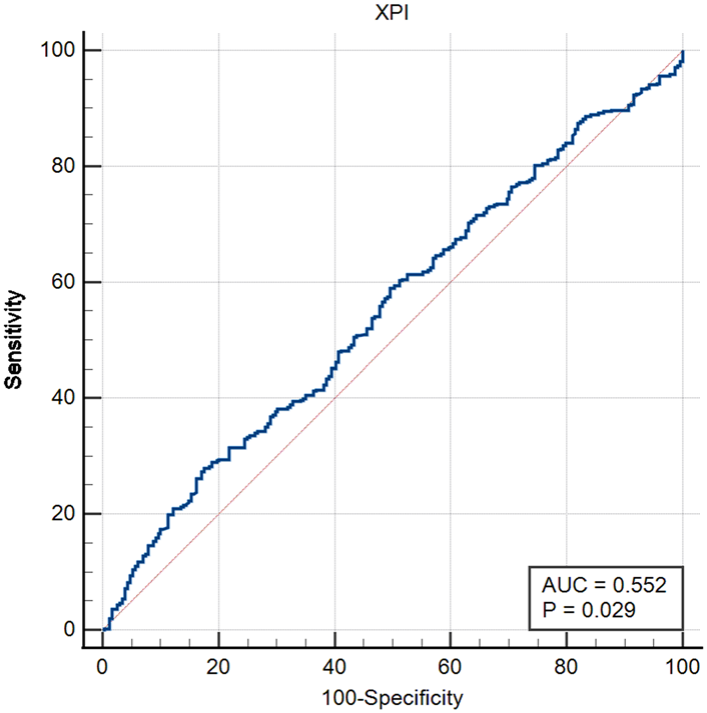
ROC curve for determination of the cut-off value of XPI for predicting 5-year OS.

Based on Fig. 4, the optimal cut-off value of XPI was 75.0, sensitivity = 0.825, specificity = 0.279, corresponding to maximum Youden index (= 0.104) for predicting 5-year OS in ROC analysis. Therefore, the patients were divided into the XPI-high (ALBI > 75) group or XPI-low (PNI ≤ 75) group.

In contrast with the XPI-low group, the XPI-high group had significantly higher 1-, 3-, and 5-year OS (87.9% vs 82.0%, 66.9% vs 54.7% and 53.7% vs 37.2%, respectively, *P* < 0.01) (Fig. 3c) and 1-, 3-, and 5-year DFS (67.6% vs 61.4% and 46.3% vs 36.2%, 35.4% and 22.9%, respectively, *P* < 0.01) (Fig. 3d). This suggests that XPI is positively correlated with survival rate in HBV-HCC patients undergoing RH.

Multivariate analysis was performed to establish the predictive models for DFS and OS of HBV-HCC patients undergoing RH. After adjustment of hazard ratio (HR) by multivariate analysis, PNI-ALBI (in Model 3) and XPI (in Model 4) could independently predict OS (all *P* < 0.01) and DFS (all *P* < 0.05) (Table 5).

**Table 5:** Prognostic models for DFS and OS by Cox multivariate regression analysis (Model 3: variables that were independent risk factors of PNI-ALBI were included; Model 4: variables that were independent risk factors of XPI were included).

Indicators	Model 3				Model 4			
	OS		DFS		OS		DFS	
	HR (95% CI)	P	HR (95% CI)	P	HR (95% CI)	P	HR (95% CI)	P
PLT	1.389 (1.108–1.741)	0.004			1.427 (1.137–1.789)	0.002	1.219 (1.012–1.469)	0.037
AFP	1.584 (1.295–1.936)	< 0.001			1.583 (1.295–1.935)	< 0.001	1.502 (1.265–1.783)	< 0.001
max-D	1.672 (1.345–2.078)	< 0.001	1.610 (1.353–1.916)	< 0.001	1.673 (1.347–2.079)	< 0.001	1.467 (1.227–1.754)	< 0.001
Number	1.365 (1.068–1.745)	0.013	1.546 (1.248–1.915)	< 0.001	1.354 (1.058–1.731)	0.016	1.586 (1.282–1.963)	< 0.001
DD	1.459 (1.195–1.782)	< 0.001	1.272 (1.074–1.508)	0.005	1.470 (1.204–1.795)	< 0.001	1.241 (1.045–1.472)	0.014
MVI	1.520 (1.230–1.877)	< 0.001	1.472 (1.223–1.771)	< 0.001	1.538 (1.244–1.901)	< 0.001	1.483 (1.231–1.787)	< 0.001
SF	1.912 (1.479–2.472)	< 0.001	1.644 (1.301–2.078)	< 0.001	1.942 (1.501–2.512)	< 0.001	1.663 (1.317–2.099)	< 0.001
PNI-ALBI	1.111 (1.030–1.198)	0.006	1.068 (1.002–1.139)	0.044				
XPI					1.460 (1.169–1.824)	0.001	1.263 (1.039–1.535)	0.019

## Discussion

Malnutrition in cancer patients is a common issue that should not be ignored. Notably, approximately 80% of solid tumor patients suffer from malnutrition during cancer treatment^22^. Preoperative malnutrition potentially leads to increased postoperative infections and complications, causing delayed recovery^23–25^. Besides, cancer cachexia caused by severe malnutrition directly causes 20% ~ 30% of cancer deaths^26^. As a digestive tract tumor, malnutrition has a significant impact on HBV-HCC prognosis.

A systemic inflammatory response is closely associated with nutritional status and affects cancer prognosis. Studies indicate that increased oxidative stress promotes cancer anorexia and cachexia^27^. Inflammation of the tumor microenvironment regulates tumor cell proliferation, invasion, and metastasis. Moreover, neutrophils and lymphocytes play a vital role in this inflammatory process. Low peripheral blood LTC may indicate a weak immune response against tumors^28^. Lymphocyte-mediated anti-tumor response regulates the prognosis of HCC patients^29^. Furthermore, lymphopenia is a poor prognostic factor for several solid tumors^30^.

In HCC prognosis, a few pro-inflammatory cytokines combined with nutritional status indicators yield unexpected results. Of note, PNI is such a combination index. Based on the ROC curve, we set the optimal cutoff value of the PNI at 46. Consequently, the 1-, 3-, and 5-year OS and DFS in the PNI-high group were significantly higher than those in the PNI-low group. This suggests that better immuno-nutrition status triggers better outcomes. Nonetheless, unlike previous studies^9,10,12^, sample size increase yields a significant difference; however, the difference is not as big as expected. We also found that the PNI-high group had higher PLT, LTC, WBC, ALB, lower TBIL, AST, HBV-DNA, AFP, max-D, DD than that of the PNI-low group.

Undoubtedly, both hepatitis B and HCC damage the liver function of patients. In other words, the liver function of patients reflects the severity of the disease to a certain extent^31,32^. As such, ALBI predicts the prognosis of HCC patients, particularly those undergoing surgery^14,32,33^. Based on the ROC curve, the optimal cutoff value of the ALBI was set at -2.80. This further confirms the previous results i.e., the ALBI-low group had a better liver function status, correspondingly, a longer OS and DFS than that of the ALBI-high group. Additionally, this paper found that the ALBI-low group had higher LTC, WBC, ALB, lower TBIL, AST, ALT, HBV-DNA, AFP, max-D, DD than that of the ALBI-high group.

Our findings confirm BCLC, PLT, AST, HBV-DNA, AFP, max-D, Number, LCI, DD, MVI, SF, PNI, and ALBI as significant prognostic factors for DFS or OS of HBV-HCC patients undergoing RH. Meanwhile, multivariate analysis verified that PLT, AFP, max-D, Number, DD, MVI, SF, PNI, and ALBI are the only independent risk factors predicting DFS or OS. Although PNI and ALBI independently predict the prognosis of HBV-HCC patients, their low accuracy and mutual exclusion as independent predictors suggest a possible combination. Based on the prognostic prediction model, the nutrition and liver function of patients are not as important as the pathological indices of the tumor. Nevertheless, they can be improved by nutritional support therapy, liver protection therapy, and immune support therapy before operation^34,35^.

This work attempted to divide patients into four subgroups. by the criteria of both. Consequently, the PNI-high + ALBI-low group had a better outcome than that of the PNI-low + ALBI-high group. However, perhaps due to insufficient sample size, the difference between them and the middle two groups was insignificant. Because of the significantly small number of patients included in group 2, (only 11 cases), there was an unexpected outcome without statistical significance. This warrants further verification in future studies.

Subsequently, PNI and ALBI were integrated to create a novel prognostic index-XPI. which was named after me. To a certain degree, XPI reflects the nutritional status, immune function, and liver function of patients. It uses ALB as the link to balance the proportion of ALB, TCL, and TBIL, hence significantly inheriting the accuracy of PNI and ALBI. An ideal cut-off value (XPI = 75) for XPI was identified by drawing the ROC curve; we confirmed XPI as an independent risk factor, predicting DFS or OS of HBV-HCC patients undergoing RH. Notably, higher XPI had a progressively better outcome. Furthermore, differences in OS and DFS between both groups were more significant than that between the PNI-high group and PNI-low group or that between the ALBI-high group and the ALBI-low group. These findings validate our hypothesis that XPI combines the advantages of PNI and ALBI to enhance the accuracy in predicting the prognosis of HBV-HCC patients undergoing RH.

Bilirubin is considered as a potential immunosuppressive toxic substance, which can lead to many immune abnormalities, including humoral immune abnormalities, imbalance of T cell subsets and abnormal production of some cytokines. Severe hyperbilirubinemia can induce apoptosis of CD4 + cells and inhibit their immune response^36^; It can also make the level of "non activated" lymphocytes decrease significantly, thus inhibiting lymphocytes and leading to immunodeficient^37^. It is well known that immunology regulations may affect cancer stage development and immunodeficient accelerates tumor progression^38^. In the current study, an obvious decrease in alpha diversity was found in the immunodeficient group mice, suggesting that this is a consequence of microbial dysbiosis, which may therefore predispose the mice to inflammatory complications and infections; meanwhile immunodeficiency may promote adaptive alterations of host gut- or tissue-based microbiome, then affect the nutritional status of mice^39^. Therefore, we believe that XPI reflects the bilirubin level, immune status and nutritional status of HBV-HCC patients. These three factors influence each other and affect the prognosis of patients together.

At the RNA level, the relationship between nutrition, immunity and cancer is also more and more mentioned. The mRNA modifications are potentially new insights into this biological basis, especially N4-Acetylcytidine on RNA expression. Jin G, et al. concluded that N4 acetylcytidine plays a key role on the cancer development, inflammation responses and immunology regulations at same time^40^. The deeper mechanism of their mutual regulation needs further study, and the potential causal effects of nutritional status on immune response and the development of HCC would also be inferred based on a large cohort under the Mendelian Randomization framework in the era of post-Genome-Wide Association Studies (GWAS) and precision medicine^41–44^.

## Conclusion

In conclusion, our findings reveal that preoperative PNI and ALBI predict the OS and DFS of HBV-HCC patients undergoing RH. Their impact on the prognosis of HBV-HCC patients is not as significant as expected, nonetheless, it cannot be ignored. XPI can precisely predict the prognosis of HBV-HCC patients undergoing RH, however, its effect warrants additional studies for validation.

## Supplementary Information

Supplementary Information.

## Data Availability

All relevant data are in the paper and Supporting Information files.
