# Author Correction: Combined prognostic nutritional index and albumin-bilirubin grade to predict the postoperative prognosis of HBV-associated hepatocellular carcinoma patients

**DOI:** 10.1038/s41598-021-95181-6

**Published:** 2021-07-28

**Authors:** Xie Liang, Xu Liangliang, Wang Peng, Yan Tao, Zhang Jinfu, Zhang Ming, Xu Mingqing

**Affiliations:** 1 Department of Liver Surgery, West China Hospital, Sichuan University, No. 37 Guo Xue Xiang, Chengdu, 610041 China; 2 Department of Hepatobiliary Surgery (2), The Affiliated Hospital of North Sichuan Medical College, Nanchong, China

Correction To: *Scientific Reports* 10.1038/s41598-021-94035-5, published online 16 July 2021

The original version of this Article contained an error in Affiliation 2, which was incorrectly given as ‘Department of Urology Hepatobiliary Surgery (2), The Affiliated Hospital of North Sichuan Medical College, Nanchong, China’. The correct affiliation is listed below:

Department of Hepatobiliary Surgery (2), The Affiliated Hospital of North Sichuan Medical College, Nanchong, China

The original Article has been corrected.

